# A Self-Powered Portable Flexible Sensor of Monitoring Speed Skating Techniques

**DOI:** 10.3390/bios11040108

**Published:** 2021-04-07

**Authors:** Zhuo Lu, Yongsheng Zhu, Changjun Jia, Tianming Zhao, Meiyue Bian, Chaofeng Jia, Yiqiao Zhang, Yupeng Mao

**Affiliations:** 1 School of Physical Education, Northeast Normal University, Changchun 130024, China; luz560@nenu.edu.cn (Z.L.); jiacf829@nenu.edu.cn (C.J.); zhangyq052@nenu.edu.cn (Y.Z.); 2 Physical Education Department, Northeastern University, Shenyang 110819, China; 2001276@stu.neu.edu.cn (Y.Z.); 2071367@stu.neu.edu.cn (C.J.); 2001264@stu.neu.edu.cn (M.B.); 3 College of Sciences, Northeastern University, Shenyang 110819, China; zhaotm@stumail.neu.edu.cn

**Keywords:** wearable sensor, self-powered, sport monitoring, speed skating

## Abstract

With the development of 5G technology, contemporary technologies such as Internet of Things (IoT) and Big Data analyses have been widely applied to the sport industry. This paper focuses on the design of a portable, self-powered, flexible sensor, which does not require an external power supply. The sensor is capable of monitoring speed skating techniques, thereby helping professional athletes to enhance their performance. This sensor mainly consists of Polyvinylidene Fluoride (PVDF) with polarization after a silvering electrode and a flexible polyester substrate. Flexible sensors are attached to the push-off joint part of speed skaters and the ice skate blade. During motion, it produces different piezoelectricity signals depending on the states of motion. The monitoring and analyzing of the real-time sensor signals will adjust the athlete’s skating angle, frequency, and push-off techniques, thus improving user training and enhancing performance. Moreover, the production of piezoelectric signals can charge the capacitor, provide power for small electronic equipment (e.g., wireless device), and extend the applications of wearable flexible sensors to the Big Data and IoT technologies in the sport industry.

## 1. Introduction

Speed skating is one of the most popular sports in the Winter Olympic Games. When skating, athletes wear Clap Skates, which need professional athletes’ high strength, endurance, and speed. In addition to personal physical fitness improvement, performance enhancement is also considered vital in the game. To enhance it, more scientific skating techniques need to be considered, such as push-off timeliness, absolute speed, skating strategies and physiological monitoring, among others. With 5G evolution, infinite biosensors are applied to the kinematics field [1,2,3,4,5,6,7,8]. With IoT technology where sensors are integrated, coaches can better understand athletes’ physical conditions, and adjust timely training programs and skating techniques to help athletes achieve better performance. At present, speed skating auxiliary sensors with training have challenges, such as huge volume, not being portable, and non-real-time data. These challenges affect the skating state of professional athletes [9,10,11]. Therefore, it is important to develop a flexible portable sensor that will overcome these challenges [12,13,14,15,16,17,18].

In recent times, the proposal of a nanogenerator has gained much attention [19,20,21,22,23,24,25,26]. This component can transform micro-chemical energy into electric energy in the environment. The environment affects the amount of produced electric energy, such as pressure, temperature, and humidity [27,28,29,30]. The manufactured sensor based on the nanogenerator does not require an external power supply or battery to provide electric energy. This is beneficial since there is no need for a battery and it is expected to be the upcoming candidate in sensor generation [31,32,33,34,35]. Therefore, by improving the materials, a flexible sensor for sport detection can be designed. It will not be influenced by temperature and humidity, and during the speed skating sport, it can be applied to monitor, in real-time, athletes’ skating techniques.

The portable, self-powered, flexible sensor that is discussed in this paper can monitor speed skating techniques without an external power supply. The sensor consists of Polyvinylidene Fluoride (PVDF) with polarization after a silvering electrode and a flexible polyester substrate. Moreover, the sensor can be easily attached to speed skating athletes’ push-off joints and ice skate blades. Based on the piezoelectric effect, the sensor produces a piezoelectric signal during its movement. The signal has sensing information that is used to analyze the skating techniques [36,37,38]. The sensor can maintain a certain output piezoelectric voltage after it is damaged. Concurrently, the produced electric energy can charge the capacitor to move the electronic devices. In practical applications, it shows that this component can transmit and receive the skating states of athletes by use of a wireless method. In this paper, our work explores more application scenarios for a wearable, flexible sensor that applies Big Data and IoT technologies in the sport industry.

## 2. Experimental

### 2.1. Sensor Fabrication

First, the PVDF power (1.5 g) was dissolved in dimethylformamide solution (8.5 g) at 50–70 °C. After vigorous stirring for 2 h, the mixture was sealed airtight and left to stand for 16 h. The PVDF film was obtained through a spin-coating process. The PVDF slurry was dropwised on a pre-cleaned Si substrate; the speed was fixed at 900 r/min and the time was fixed at 60 s. Then, the PVDF film was dried at 120 °C for 12 h. The thickness of the PVDF film can be controlled by the spin-coating speed and coating times. Secondly, both sides of the PVDF film were evaporated with Ag electrodes (300 nm). Finally, the film was polarized in an oil bath at 90 °C under a 20 kV/mm electric field. The sensor was packaged with polyester (PET) film to protect the PVDF film.

### 2.2. Characterization and Measurement

The morphology and structure of the sensor was performed by optical microscopy (SDPTOP-CX40M, Ningbo Sunny Instruments Co., Ltd, Ningbo, China.). The performance of the sensor was collected by an automatic measurement platform (containing a programmable mechanical arm and oscilloscope). A professional Olympic athlete assisted with the practical applications. Two sizes of the sensor were used to measure the sensing performance. The small-sized sensor (7 cm × 1 cm × 0.05 cm) was attached to the hip joint, and the big-sized sensor (17 cm × 2 cm × 0.05 cm) was fixed on the ice skate blade. All the tests were measured in an indoor skating rink and the temperature was kept at 14–26 °C.

## 3. Results and Discussion

**Figure 1:**
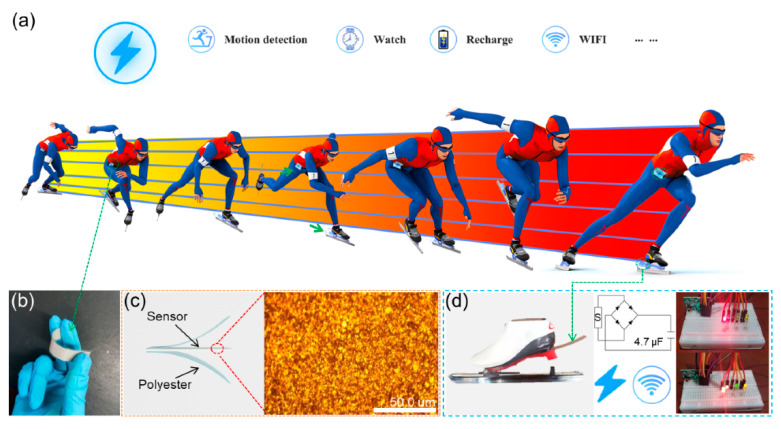
(**a**) The design of the self-powered portable flexible sensor; (**b**) optical image of the sensor; (**c**) structure and micrograph of sensor (inset); (**d**) the sensor attached to the ice skate blade (the insets show the rectifying circuit and the wireless visual panel).

The self-powered portable flexible sensor is designed to monitor the state of physiological joint changes and the track of the sliding motion in real-time. According to the signal output, a personalized sport technique improvement prescription can be made by coaches so as to adjust the athlete’s technical motions and help improve sport performance. Therefore, PVDF was chosen as the sensing material because of its fast response and high output. In the process of speed skating, the hip joint is the most representative technique, which can better reflect the athlete’s special technical level. Therefore, the sensors are designed as flexible and wearable intelligent sensors to fit on the hip joint. The package of the flexible materials greatly improves the comfort, and normal sliding is not affected. In addition, the improvement of skate blades is also one of the strategies to improve the performance of the athlete. Therefore, we attach the sensor to the skate blade to measure the correlation between the skate motion and sliding technique. Considering that the extreme motion conditions may cause damage to the sensor and some key data may become corrupted, lost or unavailable, the sensors are packed with polyester (PET). Even if the sensors have a large-area breakage, the key motion data will still output. They can better record the athlete’s sport technique in each stage of sliding, and then assist the athlete with carrying out high-level sport training. Figure 1a shows the tester wearing ice skates. The sensors are attached to the hip joint of tester and the skates, respectively. The information of the joint bending angles, motion frequencies and skate blade vibrations can be collected by sensors during skating. The whole process does not need an external power supply, and the skate blade works to charge the capacitors. It provides ideas for driving a variety of portable devices in the future, and more possibilities for motion monitoring technology. Flexible sensors can be cut and bent according to actual needs (Figure 1b). The basic structure of the sensor and its morphology under the optical microscope (inset) are shown in Figure 1c. The PVDF piezoelectric film may be easily damaged due to its flexible and thin features. We use a 0.125 mm thick polyester (PET) substrate to reinforce the PVDF piezoelectric layer. Our reinforced sensor is more durable. Figure 1d shows the sensor fixed on the root of the ice skate. During the skating process, the sensor transmits sport information in real-time (joint bending angles, motion frequencies and skate blade vibrations) and the output can be collected to charge capacitors for driving smart, wearable devices.

**Figure 2:**
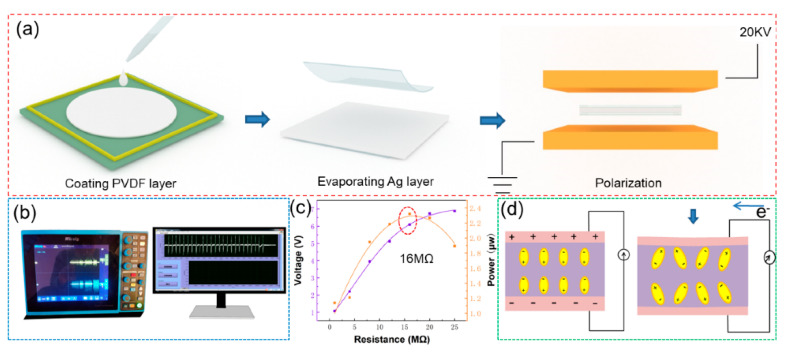
(**a**) The sensor fabrication; (**b**) measuring system; (**c**) output voltage and output power of the sensor; (**d**) working mechanism.

The progress of sensor production is shown in Figure 2a. The fabrication contains a coating PVDF layer, evaporating Ag electrodes and polarization; more details can be seen in the experimental section. The data of the sensor are collected by a sto1102c, micsig oscilloscope (origin: Shenzhen, China, Figure 2b). Figure 2c shows the output power of the sensor. With the increase in resistance, the output voltage significantly increases, and the instantaneous output power of the sensor reaches the maximum of 16 MΩ. At this time, the output piezoelectric voltage of the sensor is 6.09 V and the power is 2.32 μW. Figure 2d shows the working mechanism. When the deformation is not applied on the sensor, the dipoles in PVDF will be arranged in an ordered way, and a large number of charges will be bound on the surfaces due to the built-in electric field. When the deformation occurs, the dipole direction will be changed, and the built-in electric field will be reduced, releasing the surface bound charges [39,40]. The output signal can be detected in the external circuit. Finally, when the deformation disappears, the dipoles will return to the original state and the released charges will be rebound to the surface again. The opposite signal can be detected in the external circuit [41,42,43,44,45,46,47].

**Figure 3:**
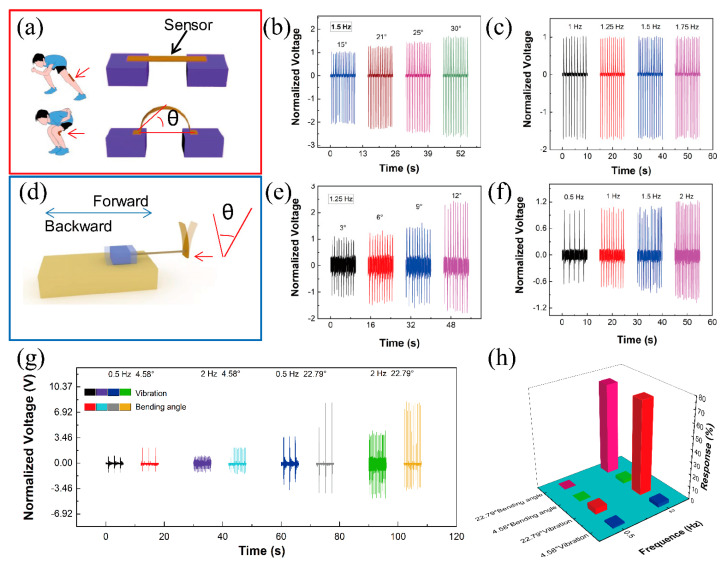
The sensing performance testing of the sensor; (**a**) the measurement system for mimicking the motion of the knee-joint; (**b**) the relationship between the output voltage and bending angles (the red line showing the fitting line); (**c**) the normalized output piezoelectric voltage against different frequencies at the same bending angles (15°); (**d**) the measurement system for mimicking the vibration on the skate blade; (**e**) the relationship between the output voltage and bending vibration (the red line showing the fitting line); (**f**) the normalized voltage against different skating frequencies at the same skating stride (3°); (**g**) the signals from two sensors (one attached to the knee-joint and another attached to the ice skate blade) for four motion states (small skating stride with low skating frequency, small skating stride with high skating frequency, big skating stride with low skating frequency and big skating stride with high skating frequency); (**h**) the relationship between motion state and the response of the sensors.

The performance of the sensor in different application scenarios is tested and analyzed in Figure 3. This sensor can be attached to the hip joint to capture the skating frequency and for mimicking the motion of joint; the deformation is applied by the stepper motor (Figure 3a). As shown in Figure S2, the response time is less than 43 ms. Figure 3b shows the output piezoelectric voltage against different joint bending angles at the same frequency (1.5 Hz). The results are treated with normalization and the baseline is the output at 15°. As the angles are 21, 25 and 30°, the piezoelectric voltage enhances 23.5, 42.1 and 65.3%, respectively. The red line is a linear fit and the linear fitting of Equation (1) is as follows:

(1) $$\frac{y=0.1951+0.01698x}{\left(r\right)=0.999}$$

where y represents the output voltage (V) and x represents the bending angles (degree). The linearity is up to 0.999. Figure 3c shows the output piezoelectric voltage against different frequencies at the same bending angles (15°). The baseline is the output piezoelectric voltage at 1 Hz. As the angles are 1.25, 1.5 and 1.75 Hz, the change of piezoelectric voltage output is less than 2%. These results show that the sensor can accurately monitor the angle change during skating. Another sensor is attached on the ice skate blade. During the skating process, the output of the sensor may be affected by the skating strides and frequencies. Figure 3d shows the measuring equipment for mimicking the vibration on the skate blade. Figure 3e shows the normalized voltage against different skating strides at the same skating frequency (1.25 Hz). As the skating stride angle increases, the hip joint increases from 3 to 6, 9 and 12°, and the normalized voltage is 0, 17.5, 43.5 and 133.9 Hz, respectively. The red line is a linear fit, and the linear fitting of Equation (2) is as follows:

(2) $$\frac{y=0.41795+0.1426x}{\left(r\right)=0.92757}$$

where y represents the output voltage (V) and x represents the vibration (degree). The linearity is up to 0.92757. Figure 3f shows the normalized voltage against different skating frequencies at the same skating stride (3°). As the skating frequency increases from 0.5 to 2 Hz, the normalized voltage is 0, 2.3, 3.6 and 15.4 Hz, respectively. Figure 3g shows the signals from two sensors (one attached to the knee-joint and another attached to the ice skate blade) for four motion states (small skating stride with low skating frequency, small skating stride with high skating frequency, big skating stride with low skating frequency, and big skating stride with high skating frequency). When the skating state is small skating stride with low skating frequency, the output piezoelectric voltages of the sensors are 1.12 V (hip joint) and 2.4 V (skating state), respectively. When the skating state is small skating stride with high skating frequency, the output piezoelectric voltages of the sensors are 1.15 V (hip joint) and 2.38 V (skating state), respectively. When the skating state is big skating stride with low skating frequency, the output piezoelectric voltages of the sensors are 4.17 V (hip joint) and 9.21 V (skating state), respectively. When the skating state is big skating stride with high skating frequency, the output piezoelectric voltages of the sensors are 4.67 V (hip joint) and 9.17 V (skating state), respectively. Figure 3h shows the relationship between the motion state and the response of the sensors. The response of the sensor can be calculated from the following equation:

(3) $$R\%=\left|\frac{V_{0}-V_{i}}{V_{i}}\right|\times 100\%$$

where V_0_ represents the output piezoelectric voltage at 1.12 V. Sensors can be attached on the joints and equipment to monitor the joint angle, motion frequency and other information of the tester in the skating process. Via this information, we can correct the speed skating techniques, improving the athlete’s sport performance.

**Figure 4:**
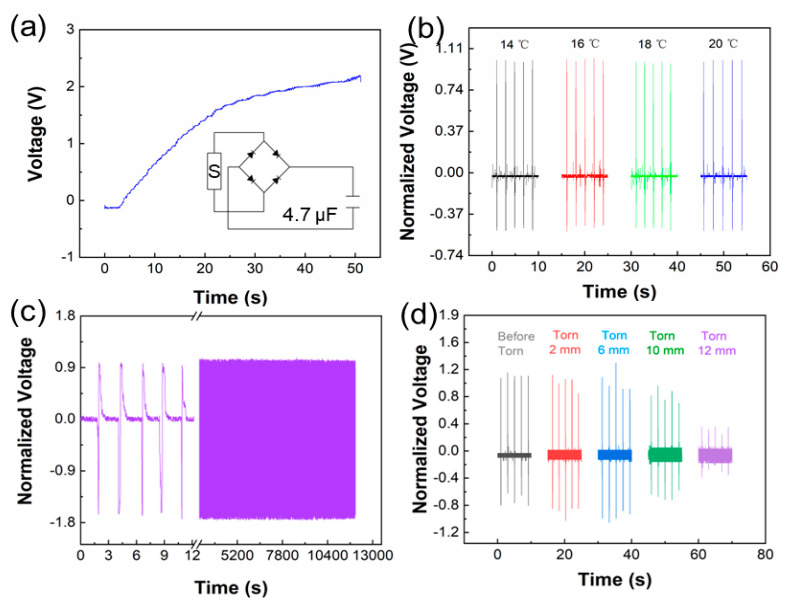
(**a**) Charging a 4.7 μf capacitor through a rectifying bridge; (**b**) output piezoelectric voltage of sensor at different temperatures; (**c**) durability of the sensor for 3.5 h; (**d**) the output piezoelectric voltage after cutting the sensor.

As shown in Figure 4a, the sensor is connected to a 4.7 μf capacitor through a rectifying bridge. The capacitor can be charged to 2.20 V in 50 s, and the charged capacitor can drive other portable devices, such as Bluetooth and WiFi. Faster motion frequencies and larger bending angles can shorten the charging time. Figure 4b shows the output piezoelectric voltage of the sensor in a normal speed skating temperature environment (Figure S1a). The temperatures of Olympic venues for speed skating are between 16 to 20 °C and the test temperature is kept at 14–20 °C. With the temperature ranging from 14 to 20 °C, the normalized voltage changes less than 2%. The sensors are not affected by temperatures and can work well for skaters. In addition, the durability of the sensor has been tested. Under the same working frequency, the output piezoelectric voltage is stable for 3.5 h (Figure 4c). A high-intensity and fierce skating process may damage the flexible sensor. Therefore, we cut the sensor to test the performance (Figure S1b). Figure 4d shows the output piezoelectric voltage after cutting the sensor. The results show that the sensor can maintain over 90% of the output piezoelectric voltage after cutting 6 mm, and the sensor can still maintain about 30% output piezoelectric voltage after cutting 12 mm. Compared with other works, the self-powered portable flexible sensor has higher output (Table S1). The sensor can continually work under extreme conditions. The sensor still maintains 74% of the initial output voltage in 2000 s, even if the damage is up to 65% (Figure S1c). Details of the output are shown in Figure S1d. Moreover, the output electrical energy can charge capacitors for driving other smart, wearable devices.

**Figure 5:**
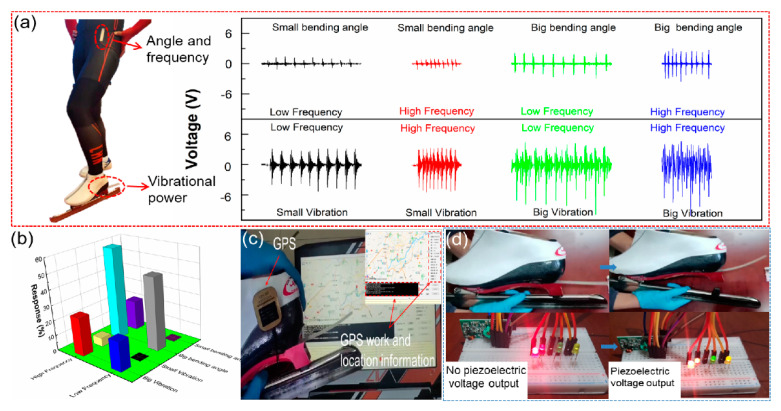
(**a**) Output piezoelectric voltage of speed skating during four sport states; (**b**) response of the sensor; (**c**) position information recorded by GPS system; (**d**) a simple wireless system for monitoring motion state.

The speed skating performance depends on three major techniques: start action, straight line speed and curve skill. As shown in Figure 5a, the sensors are attached to the hip joint and ice skate blade, respectively. Under four sport states, the output is shown in the inset. Figure 5b shows the responses of the sensor for four sport states. These results show that the signal can be real-time recorded and accurately reflect the sport information (joint bending angles, motion frequencies and skate blade vibrations), assisting athletes in correcting speed skating techniques immediately. During the skating process, the self-powered sensor can charge the capacitor (Figure S3). A 4.7 μf capacitor can be charged to 1.32 V in 50 s. Figure 5c shows that when the capacitor is charged to 5 V, the GPS can be driven to transmit signals, and the position information can be recorded (Video S1). A real-time monitoring system for athletes is shown in Figure 5d. The system consists of the self-powered portable sensor, a wireless transmitter and a visual panel. The piezoelectric signal of the sensor can be transmitted by wireless transmitter. The times and the numbers of LEDs on the visual panel, which are lit up, can reflect the sport states (Video S2). This system can help coaches and athletes quickly find technical weaknesses and improve their skating skills.

## 4. Conclusions

In conclusion, this study proposed a type of self-powered flexible sensor that is used to monitor skating states in real-time. The sensor can be easily attached to the tester’s body surface joint or commonly-used equipment to help monitor sport performance. With the piezoelectric effect, this sensor can collect the human body’s kinetic energy during its motion. Moreover, it can produce a piezoelectric signal that contains sensing information while the sensor is in the moving state. During the piezoelectric signals analysis, the professional athletes are able to adjust their training programs in real-time. The production of Piezoelectric signals can also charge the capacitor to drive small electronic devices. In practical applications, the experiment of a wireless transmitting piezoelectric signal exhibited the potential for this self-powered flexible sensor. This study has extended the applications of wearable flexible sensors that use Big Data and IoT technologies in the sport industry.

## Data Availability

The data presented in this study are available in supplementary material.

## Supplementary Materials

The following are available online at https://www.mdpi.com/article/10.3390/bios11040108/s1, Figure S1: The performance against different conditions, Figure S2: Response time of the self-powered portable flexible sensor, Figure S3: Charging a capacitor, Table S1: The self-powered portable flexible sensor in comparison with previous works, Video S1: GPS driven by the self-powered portable flexible sensor, Video S2: Numbers of LEDs driven by the self-powered portable flexible sensor.
