# Supplementary material for: A Self-Powered Portable Flexible Sensor of Monitoring Speed Skating Techniques
Source: Biosensors (Basel). 2021 Apr 7;11(4):108. doi: 10.3390/bios11040108 (PMC8067624; doi:10.3390/bios11040108)
Supplement: Supplementary file 1 [file biosensors-11-00108-s001.zip › biosensors-1143405-supplementary/biosensors-1143405-SM/biosensors-1143405.docx]

A Self-Powered Portable Flexible Sensor of Monitoring Speed Skating Techniques

Zhuo Lu ^1^, Yongsheng Zhu ^2^, Changjun Jia ^2^, Tianming Zhao ^3^, Meiyue Bian ^2^, Chaofeng Jia ^1^, Yiqiao Zhang ^1^ and Yupeng Mao ^1,2,^*

| **Citation:** Lu, Z.; Zhu, Y.; Jia, C.; Zhao, T.; Bian, M.; Jia, C.; Zhang, Y.; Mao, Y. A Self-Powered Portable Flexible Sensor of Monitoring Speed Skating Techniques. *Biosensors* **2021**, *11*, 108. https://doi.org/10.3390/ bios11040108  Received: 26 February 2021  Accepted: 1 April 2021  Published: 7 April 2021  **Publisher’s Note:** MDPI stays neutral with regard to jurisdictional claims in published maps and institutional affiliations.  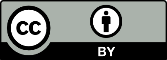  **Copyright:** © 2021 by the authors. Licensee MDPI, Basel, Switzerland. This article is an open access article distributed under the terms and conditions of the Creative Commons Attribution (CC BY) license (http://creativecommons.org/licenses/by/4.0/). |
| --- |

^1^ School of Physical Education, Northeast Normal University, Changchun 130024, China; luz560@nenu.edu.cn (Z.L.); jiacf829@nenu.edu.cn (C.J.); zhangyq052@nenu.edu.cn (Y.Z.)

^2^ Physical Education Department, Northeastern University, Shenyang 110819, China; 2001276@stu.neu.edu.cn (Y.Z.); 2071367@stu.neu.edu.cn (C.J.); 2001264@stu.neu.edu.cn (M.B.)

^3^ College of Sciences, Northeastern University, Shenyang 110819, China; zhaotm@stumail.neu.edu.cn

***** Correspondence: maoyupeng@pe.neu.edu.cn

**Table S1.** The self-powered portable flexible sensor in comparison with previous works.

|  | **The power supply mode** | **Output Voltage** | **Work after Damage** | **Response** | **Reference** |
| --- | --- | --- | --- | --- | --- |
| Our work | piezoelectric | 0–0.64 + | Yes | 0–70% |  |
| Other 1 | piezoelectric | 0–0.6 + | No | 0–30% | [23] |
| Other 2 | piezoelectric | 0–0.18 + | No | 0–250% | [24] |
| Other 3 | triboelectric | 0–20 + | No | None | [47] |


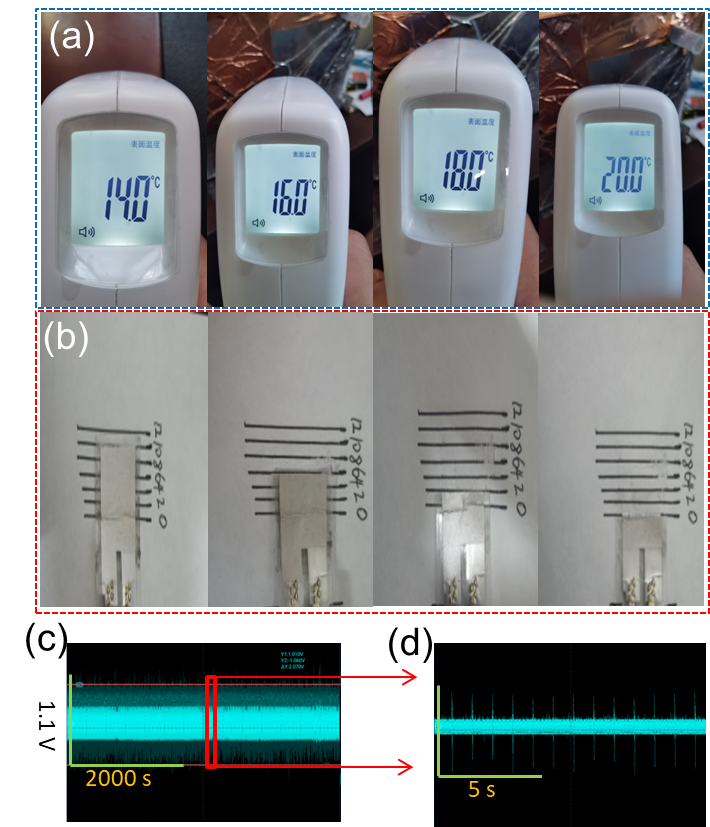


**Figure S1.** The performance against different conditions. (**a**) Operating temperature of sensor; (**b**) optical image processing in sensor segmentation; (**c**) the output piezoelectric voltage after 65% damage; (**d**) details of the output piezoelectric voltage.


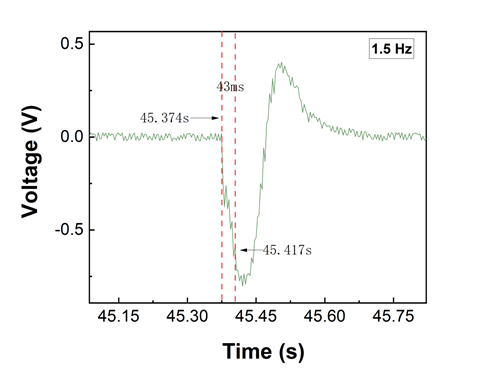


**Figure S2.** Response time of the self-powered portable flexible sensor.





**Figure S3.** Charging a capacitor.

**Supplementary Video S1.** GPS driven by the self-powered portable flexible sensor. The GPS can be driven to transmit signals, and the position information can be recorded.

**Supplementary Video S2.** Numbers of LEDs driven by the self-powered portable flexible sensor. The times.
